# Combined Phosphorus and Potassium Starvation Upregulates Sodium Accumulation by HKT1;1 in Tomato Plants

**DOI:** 10.1111/pce.70715

**Published:** 2026-07-03

**Authors:** Jesús Amo, Adela Belchí, Elisa Jiménez‐Estévez, Adrián Yáñez, Maria José Asins, Andrés Belver, Vicente Martínez, Francisco Rubio, Manuel Nieves‐Cordones

**Affiliations:** ^1^ Plant Nutrition Department, CEBAS‐CSIC Campus de Espinardo Murcia Spain; ^2^ Instituto Valenciano de Investigaciones Agrarias (IVIA) Valencia Spain; ^3^ Department of Stress, Development and Signalling of Plants, Estación Experimental del Zaidín Consejo Superior de Investigaciones Científicas (EEZ CSIC) Granada Spain

## Abstract

Na^+^ can substitute K^+^ under K^+^ deficiency conditions, which is denominated as beneficial Na^+^. Despite its relevance at the physiological level and its interest in agriculture, the mechanisms allowing its uptake and use by plants remain unclear. The accumulation of beneficial Na^+^ has been associated with K^+^ deficiency, but the deficiency of other mineral nutrients could also affect this process. In this study, we observed that the combined deficiency of K^+^ and P (−K −P) resulted in a 2‐fold increase in the root Na^+^ content in tomato plants with respect to the single K^+^ deficiency (−K). Such a response was observed also in quinoa and barley but not in Arabidopsis, wheat, rice, soybean and *Solanum pennellii*, evidencing natural variability for this trait. Pi uptake in −K −P plants was not coupled to Na^+^ as deduced from electrophysiology and plant growth experiments. Finally, we show that the Na^+^ channel HKT1;1, but not HKT1;2, contributes to Na^+^ uptake in tomato plants, in particular, in the −K −P condition where a 40‐45% reduction in root Na^+^ content is observed in *HKT1;1*‐silenced lines. Therefore, the information acquired on HKT1;1 is crucial for boosting Na^+^ uptake in plants.

## Summary statement

The combined deficiencies of K^+^ and P stimulates Na^+^ accumulation in tomato, barley and quinoa plants. The Na^+^ channel HKT1;1, but not HKT1;2, contributes to Na^+^ accumulation in tomato plants, in particular, under K^+^ and P deficiencies.

## 1 Introduction

Sodium (Na^+^) is well known as a toxic cation for plants when present at high concentrations. However, Na^+^ can be beneficial at low external concentrations (low mM to µM range) when potassium (K^+^) is scarce. Na^+^ can replace K^+^ to some extent in cellular functions, such as osmotic adjustment, regulation of cell membrane potential and negative charge balance (Wakeel et al. 2011). Plants exhibit variability in the use of beneficial Na^+^ (Ma et al. 2023). For example, in sugar beet or turnip plants, Na^+^ can substitute K^+^ to a large extent, whereas in rye or soybean plants such a substitution is not observed (Ma et al. 2023). The intrinsic capacity of plants to use beneficial Na^+^ is of great interest for agriculture as Na^+^ fertilisers are cheaper, and have less CO_2_ footprint than the K^+^ fertilisers and Na^+^ improves the taste of the edible parts of certain crops (Thorne and Maathuis 2022). Contrarily to the large body of information on the physiological aspects of beneficial Na^+^ (Thorne and Maathuis 2022; Ma et al. 2023), the molecular mechanisms involved are poorly understood. One of the critical steps to use of the benefits of Na^+^ is its uptake from the soil solution, a process mediated by membrane transport proteins. In rice, the Na^+^ channel OsHKT2;1 is the major component of Na^+^ uptake in K^+^‐starved plants (Horie et al. 2007), which is the only transport system identified so far mediating beneficial Na^+^ uptake. In tomato plants, the CBL‐interacting protein kinase CIPK23 is a major regulator of Na^+^ uptake as *cipk23* plants barely accumulate Na^+^ under K^+^‐limiting conditions (Amo et al. 2021). This mutant also served as a tool to evaluate the relevance of Na^+^ in K^+^‐starved tomato plants. It was shown that, under low K^+^ supply, *cipk23* roots were longer and lighter than the WT ones and such effect was reversed by adding 10 mM Na^+^ (Amo et al. 2024). CIPK23 together with the Calcineurin B‐like proteins CBL1/9 activate the K^+^ transport systems LKT1 and HAK5 to mediate root K^+^ uptake in tomato plants (Amo et al. 2021). Besides its role in root K^+^ uptake, the tomato CIPK23 may be a positive regulator of several Na^+^ transport systems, among which HAK/KUP/KT and HKT transport systems are likely candidates (Amo et al. 2024). In addition to the transport systems regulated by CIPK23, other transport systems mediate Na^+^ uptake in tomato roots which form a CIPK23‐independent Na^+^ transport pathway, which is sensitive to Ca^2+^, La^3+^, Ba^2+^ and Li^+^ (Amo et al. 2024). Interestingly, unlike tomato plants, Arabidopsis plants have a limited capacity to accumulate Na^+^ under K^+^ deficiency and CIPK23 does not play a role in this process (Amo et al. 2021). Therefore, it is important to perform studies on crops as relevant physiological mechanisms are absent in Arabidopsis (Nieves‐Cordones and Rubio 2025).

In tomato, there are two genes encoding for HKT channels, *HKT1;1* and *HKT1;2* (Asins et al. 2013). Both genes are closely located in the genome and associated with a major Quantitative Trait Locus (QTL) responsible for modulating Na^+^/K^+^ homoeostasis in shoots under saline conditions (Asins et al. 2013). Both tomato HKT channels present high selectivity for Na^+^ over K^+^ and other cations (Asins et al. 2013; Almeida et al. 2014). Studies carried out with RNAi‐silenced lines for the *HKT* genes showed that HKT1;2 is a major actor in salt tolerance by mediating the unloading of Na^+^ from xylem vessels whereas HKT1;1 plays a minor role in this context (Jaime‐Pérez et al. 2017; Romero‐Aranda et al. 2020; Asins et al. 2022). It is worth to highlight that the tomato HKT1;2 and its Arabidopsis homologue AtHKT1;1 prevent massive Na^+^ accumulation in reproductive tissues under salt stress, which allowed plants to reduce the yield penalty caused by salinity (Romero‐Aranda et al. 2020; Uchiyama et al. 2023). In K^+^‐deficient tomato plants, proof of the contribution of HKT1;1 and HKT1;2 to Na^+^ uptake *in planta* is currently pending.

Mineral nutrient stress, either deficiency or excess, is perceived by plant cells, but the information about a given nutrient stress is not decoded alone (Bouain et al. 2019; DeLoose et al. 2024). Plants can integrate several nutrient availability signals to produce a given response, which in some cases cannot be anticipated from the sum of each individual nutrient stress (Bouain et al. 2019; DeLoose et al. 2024). Therefore, studies on specific combinations of mineral nutrient stresses are needed to understand the major outcome of nutrient interactions. Cross‐talk between mineral anion and cation transport is well documented and involve coregulation of the corresponding transport systems (Nieves‐Cordones et al. 2019; Raddatz et al. 2020). Their joint requirement for the plant stems from their charge complementarity and their cooperative effect on osmotic adjustment (Hawkesford et al. 2023). Thus, it is not surprising that anions such as NO_3_^‐^ require certain amounts of K^+^ to be efficiently transported throughout the plant (Raddatz et al. 2020). However, excess of K^+^ can be detrimental for anion homoeostasis as in the case of Pi (Ródenas et al. 2019). With respect to Na^+^, its interaction with mineral nutrients has been mainly studied under salinity conditions (sodium excess), but not at low Na^+^ concentrations (range for beneficial Na^+^) (Nieves‐Cordones et al. 2019; Raddatz et al. 2020; DeLoose et al. 2024). Part of the Na^+^‐nutrient interactions originate from coordinated transport through the same membrane protein as it has been proposed for NO_3_^‐^ xylem load in Arabidopsis (Álvarez‐Aragón and Rodríguez‐Navarro 2017). In the case of *Zostera marina*, electrophysiological evidence supports the existence of Na^+^/NO_3_^‐^ and Na^+^/PO_4_^3‐^ cotransport mechanisms in leaves (García‐Sánchez et al. 2000; Rubio 2005). Recently, it was shown that ZmNRT2;1 operated as a Na^+^/NO_3_^‐^ symporter in which Na^+^ allows uphill NO_3_^‐^ transport (Rubio et al. 2023). Despite the information presented above, the interactions of Na^+^ at low external concentrations with anions have not been studied.

In the present study, we show that Na^+^ accumulation is enhanced by the combined deficiency of K^+^ and P in tomato plants growing in the presence of micromolar concentrations of Na^+^ concentration (90 µM). This capacity is present in other plant species, such as quinoa or barley, but not in others such as Arabidopsis or soy. Although P deficiency is necessary to stimulate Na^+^ accumulation, such response is not related with P uptake. Lastly, experiments with RNAi‐silenced lines demonstrated that HKT1;1 contributes to Na^+^ accumulation at low external concentrations in tomato plants, in particular, under combined deficiency of K^+^ and P.

## 2 Materials and Methods

### 2.1 Plant Material and Growth Conditions

Tomato plants (*Solanum lycopersicum* L. cv. Micro‐Tom), *S. pennellii* (acc. PE47), *Chenopodium quinoa* (cv. Titicaca), *Glycine max* (cv. Mungo), *Hordeum vulgare* (cv. Pewter), *Triticum aestivum* (cv. Califa sur), *Oryza sativa* (cv. Nipponbare) and *Arabidopsis thaliana* (ecotype Col‐0) were used in this study as wild‐type plants (WT). Additionally, *cipk23* tomato mutant plants (cv. Micro‐Tom) (Amo et al. 2021) and the near‐isogenic line NIL14, carrying the *HKT1;1* and *HKT1;2* genes from *S. galapagense* in homozygosis (Asins et al. 2013) while most of its genome belongs to *S. lycopersicum* var. Cerasiforme (Asins et al. 2022), as well as its corresponding *HKT1;1 and HKT1;2* single and double silenced RNAi lines (Jaime‐Pérez et al. 2017; Romero‐Aranda et al. 2021) were used.

Hydroponic culture was performed on plastic containers with aerated 1/5 Hoagland solution that consisted of macronutrients (mM): 1.4 KCl, 1.4 Ca(NO_3_)_2_, 0.35 MgSO_4_ and 0.1 Ca(H_2_PO_4_)_2_ and micronutrients (μM) 50 CaCl_2_, 12.5 H_3_BO_3_, 1 MnSO_4_, 1 ZnSO_4_, 0.5 CuSO_4_, 0.1 H_2_MoO_4_, 0.1 NiSO_4_ and 10 Fe‐EDDHA. All plants except Arabidopsis were grown in a controlled environment growth chamber with 16/8 h light/dark photoperiod. At 25°C(day), 20°C (night) temperature, 65% relative humidity and 360 μmol m^−2^ s^−1^ flux density. pH of the nutrient solution was adjusted daily to 5.5. The growth solution was renewed every week. To obtain −K solutions, KCl was not added. To obtain –N solutions, Ca(NO_3_)_2_ was not added and CaCl_2_ was added instead to reach 1.55 mM Ca^2+^. To obtain –S solutions, MgSO_4_ was not added and MgCl_2_ was added instead to reach 0.35 mM Mg^2+^. To obtain −P solutions, Ca(H_2_PO_4_)_2_ was not added and CaCl_2_ was added instead to reach 1.55 mM Ca^2+^. To obtain −K −P solutions, KCl and Ca(H_2_PO_4_)_2_ were not added and CaCl_2_ was added instead to reach 1.55 mM Ca^2+^. The Na^+^ concentration in hydroponic culture solutions was 90 μM as measured using an ICP‐OES equipment. Arabidopsis plants were germinated in cut Eppendorf tubes filled with rockwool in 2 L containers and grown in the same hydroponic solutions as described above. Arabidopsis growth conditions were with an 8/16‐h light/dark cycle at 150‐μmol m^−2^ s^−1^ flux density, 22°C and 65% relative humidity. All plants, except Arabidopsis, were grown in full nutrient solution in hydroponics during 14 days. Then, plants were subjected to single or combined nutrient deficiencies for 7 days as indicated. Arabidopsis were grown in full nutrient solution in hydroponics during 30 d and starved for K^+^ and/or P for 7 days.

### 2.2 Ion Content Determination in Plant Tissues

To quantify the ion content, plant organs were collected at the end of the experiments and their fresh weight was determined. The dried plant material was subjected to microwave‐assisted digestion in a 5:3 (vol:vol) HNO_3_ solution using a CERM MarsXpress system (Mathews, NC, USA). Cation content was then analysed via inductively coupled plasma mass spectrometry (ICP‐MS) using an Iris Intrepid II ICP spectrometer (Thermo Electron Corp., Franklin, MA, USA).

### 2.3 Determination of Plasma Membrane Potentials in Root Cells

Single electrodes were used to measure changes in cell membrane potential after Na^+^ or Pi treatments as indicated. To measure Na^+^‐induced depolarisations, WT plants were grown for 7 days under −K or −K −P treatments. Two‐cm root tips were excised and mounted on a perfusion chamber. Single‐barrelled borosilicate microelectrodes were filled with 0.2 M KCl and used to impale root epidermal or cortical cells. A solution containing 0.5 mM CaCl_2_ buffered to pH 6 (10 mM MES/Bis‐Tris) was perfused (Amo et al. 2021). 1 mM Na^+^ was added to the external solution as NaCl. Membrane potential values were recorded using a 773 WPI high‐impedance amplifier connected to a Digidata 1440B (Molecular Devices, UK) and visualised in a computer with the Axoscope software (Molecular Devices, UK).

To measure Pi‐induced membrane depolarisations in root epidermal and cortex cells, *cipk23* mutant tomato plants grown for 7 days in −K −P solution were used to reduce Na^+^ uptake by CIPK23‐regulated transport systems. In these experiments, the pH of the perfusion solution was adjusted to 7.2 with 10 mM HEPES, instead of using MES, to reduce the activity of H^+^/Pi symporters. Membrane depolarisations were induced by adding 0.2 mM Pi (added as Ca(H_2_PO_4_)_2_) to root cells under two different levels of Na^+^: 1) no added Na^+^ (0Na0.2P) or 2) 1 mM Na (1Na0.2 P) and data were recorded and analysed as described above. Transition from 10 mM MES/Bis‐Tris solution pH 6 to 10 mM HEPES pH 7.2 did not entail significant changes in the membrane potential.

### 2.4 qRT‐PCR

Total RNA was extracted from Micro‐tom WT roots using the NucleoSpin RNA Plant Kit (Macherey‐Nagel, Düren, Germany). To eliminate residual DNA contamination, RNA samples were treated with the DNA‐free Kit (Thermo Fisher Scientific, Waltham, MA, USA). Subsequently, cDNA was synthesised using the High Capacity cDNA Reverse Transcription Kit (Thermo Fisher Scientific). Then, quantitative RT‐PCR (qPCR) was performed on a 7500 Real‐Time PCR System (Thermo Fisher Scientific) and reagents from FastGene Probe 2x qPCR Universal Mix (Nippon Genetics, Japan). Relative gene expression levels were normalised to the endogenous reference gene *EF1α* (encoding elongation factor 1‐alpha) and calculated as 40 − ΔCt (ΔC_t_ = C_t_^target^ − C_t_^EF1α^) (Nieves‐Cordones et al. 2023). The following primers were used in qRT‐PCR assays: HKT1;1‐F 5′ TCTAGCCCAAGAAACTCAAAT 3′, HKT1;1‐R 5′ CTAATGTTACAACTCCAAGGAATT 3′, HKT1;2‐F 5′ TCCAGATGGTCATTGCAAGGA 3′, HKT1;2‐R 5′ GCCTTGCCACCTCTTTGATT 3′, EF1α‐F 5′ GGCGGTGGCGAGCAT 3′, and EF1 α‐R 5′ AAACCAAGGCACCTCAACAAA 3′.

## 3 Results

### 3.1 Combined K^+^ and P Deficiencies Enhance Na^+^ Accumulation in Tomato but not in Arabidopsis

With the aim of assessing whether certain anions exerted an effect on Na^+^ uptake in K^+^‐deficient tomato plants, we grew Micro‐tom wild‐type (WT) plants under full nutrient solution in hydroponics for 14 d at an external Na^+^ concentration of 90 µM. Then, plants were subjected to combined deficiency of K^+^ (−K) with that of NO_3_^−^ (−K −N), PO_4_^3‐^ (−K −P) and SO_4_^2−^ (−K −S). A group of plants grown under full nutrient solution was kept as a control. As expected, K^+^‐deficient plants exhibited higher Na^+^ content in roots compared with +K plants. A modest increase in shoot Na^+^ accumulation due to K^+^ deficiency was also observed (Figure 1a). Regarding the combined deficiencies of K^+^ and anions and, compared with −K plants, the −K −P treatment produced an important increase in root Na^+^ content (2.65‐fold increase), the −K −N treatment had no effect and the −K −S treatment resulted in a reduction in root Na^+^ content (30%) (Figure 1a). To assess whether the positive effect of the −K −P treatment on Na^+^ content (Figure 1a) was due to increased Na^+^ permeability at the plasma membrane of root cells, we used single electrodes to measure Na^+^‐induced membrane depolarisations in root cortex cells. The resting membrane potentials recorded in root cortex cells were −154 ± 4 mV and −151 ± 2 mV in −K and −K −P roots, respectively. When 1 mM Na^+^ was added to the external solution, the membrane potential was depolarised by 2.7 mV in −K root cortex cells (Supporting Information Figure S1). However, in −K −P root cortex cells, 1 mM Na^+^ depolarised the membrane potential by 4.3 mV (Supporting Information Figure S1). Therefore, Na^+^ permeability was enhanced in −K −P root cortex cells, suggesting that a Na^+^ transport system was upregulated under these conditions. Analysis of the K^+^ content in these plants showed that the increase in Na^+^ content due to the −K −P treatment with respect to the −K treatment was not accompanied by a change in the K^+^ content (Supporting Information Figure S2a). Such a positive effect of the −K −P treatment on root Na^+^ content was unexpected. Compensation of P deficiency with Na^+^ (a cation) cannot be explained by charge compensation, as it happens with K^+^ and Na^+^ (Figure 1) or K^+^ and Mg^2+^ (Amo et al. 2021), because P deficiency involves a reduction in negative charges in plant cells and Na^+^ accumulation provides positive ones. Therefore, we further studied the positive effect of the −K −P treatment on Na^+^ content. In a following experiment, we subjected tomato plants to single and combined K^+^ and P deficiencies to assess whether the increase in root Na^+^ content was induced by the single P deficiency or by the combination of both K^+^ and P deficiencies. The growth schedule was similar to the experiment shown in Figure 1a (14 days with full nutrient solution followed by 7 days of nutrient deficiencies). It was observed that only the combined deficiency of K^+^ and P gave rise to a high root Na^+^ content, whereas P deficiency alone had no significant effect on this parameter (Figure 1b). Determination of dry weights revealed that the higher root Na^+^ content in −K −P plants compared to −K plants was not associated with a higher root dry weight (Supporting Information Figure S2c). However, −K −P roots showed a higher water content than −K roots (Supporting Information Figure S2d), which could be due to the positive osmotic effect of Na^+^ that permitted the retention of more water in the roots. Then, we wondered whether the positive effect of the −K −P treatment on Na^+^ accumulation was present in Arabidopsis. Thus, a similar experiment to that shown in Figure 1b was conducted with Arabidopsis WT plants involving single and combined K^+^ and P deficiencies. Similar Na^+^ content was observed for roots and shoots under the four treatments studied (Figure 1c). Therefore, Arabidopsis lacked the Na^+^ accumulation response induced by the combined K^+^ and P deficiency observed in tomato.

**Figure 1:**
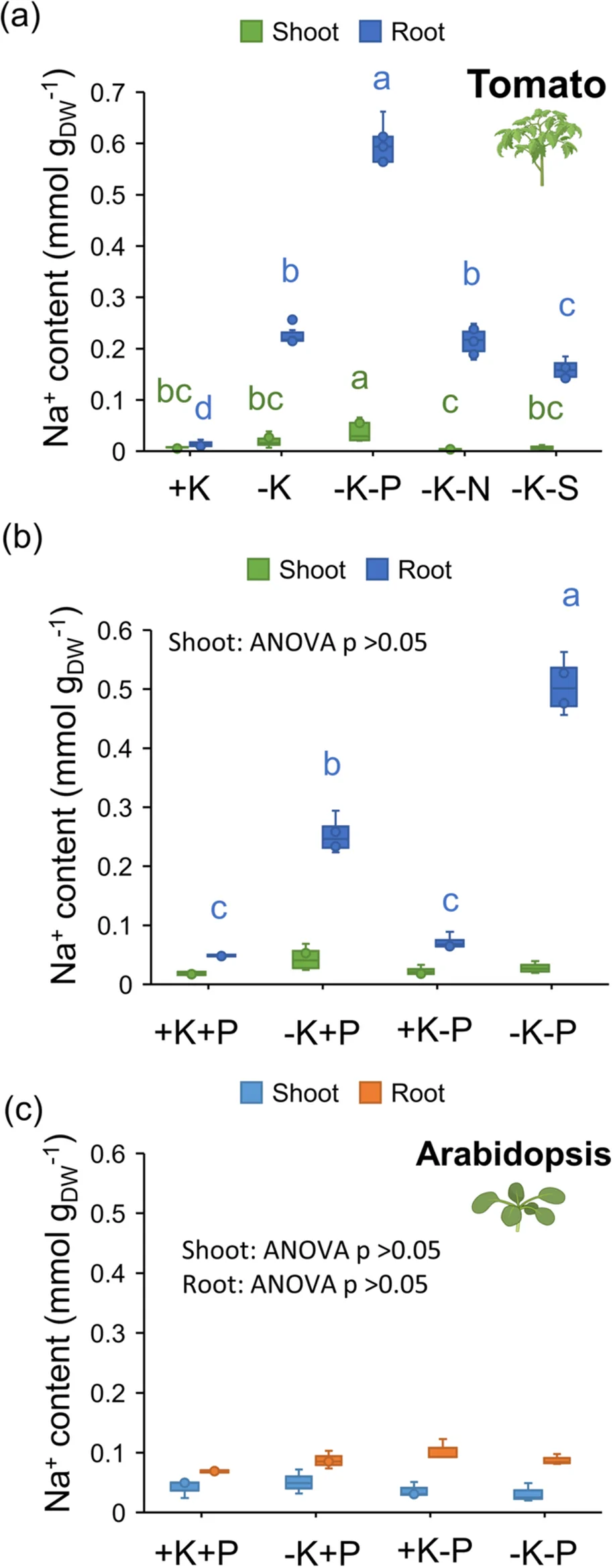
Na^+^ accumulation is enhanced in tomato plants subjected to combined K^+^ and P deficiencies. (a) Na^+^ content in roots and shoots of wild‐type (WT) tomato plants subjected to K^+^ starvation combined with different anions deficiencies (P, N or S). Plants were grown in full nutrient solution in hydroponics during 14 days. Then, plants were subjected to combined deficiency of K^+^ (−K) with that of NO_3_^−^ (−K −N), PO_4_^3−^ (−K −P) and SO_4_^−^ (−K −S) for 7 days. The external Na^+^ concentration in all solutions was 90 µM. (b) Na^+^ content in root and shoots of WT tomato plants grown under individual or combined K^+^ and P deficiencies. (c) Na^+^ content in root and shoots of WT Arabidopsis plants grown under individual or combined K^+^ and P deficiencies for 7 d. Data are presented in boxplots (*n* = 4–6 for tomato, *n* = 3 for Arabidopsis). Letters represent different groups of data at *p* < 0.05 based on LSD's post hoc test following ANOVA. Icons were obtained from biorender.com.

### 3.2 Combined K^+^ and P Deficiencies Enhance Na^+^ Accumulation in *C. quinoa* and *H. vulgare*

Since we did not find other reports showing the positive effect of K and P deficiencies on Na^+^ accumulation at low external concentrations, we conducted a series of experiments with different plant species to check to what extent such a response was conserved among plants. These experiments included the following plant species: *Solanum pennellii (wild tomato)*, *Chenopodium quinoa*, *Glycine max*, *Hordeum vulgare*, *Triticum aestivum*, and *Oryza sativa*. The four treatments described in Figure 1b were used in this experiment (+K, −K, −P and −K −P) (Figure 2). Interestingly, these plants showed a large diversity in the response not only to the −K −P treatment but also to the −K treatment in terms of Na^+^ accumulation in plant tissues. Despite Na^+^ content increasing in all species subjected to the −K treatment compared to the +K treatment, the extent to which Na^+^ was accumulated in roots and shoots was different. With respect to −K roots, root Na^+^ content ranged from low values in *G. max* (about 0.08 mmol g_DW_^−1^) (Figure 2c) to high values in *C. quinoa* (about 0.7 mmol g_DW_^−1^) (Figure 2b). *H. vulgare* plants exhibited high Na^+^ content in the shoots (about 0.4 mmol g_DW_^−1^), which was from 4‐ to 20‐fold higher than the shoot Na^+^ content of the other plant species analysed. Regarding the effect of the −K −P treatment on Na^+^ content compared to the −K treatment, the plant species under study also behaved differently. In *C. quinoa* and *H. vulgare*, root Na^+^ content increased due to the −K −P treatment (Figure 2b,d), in *S. pennellii* and *O. sativa* it did not change (Figure 2a,f) and in *G. max* and *T. aestivum* it was reduced (Figure 2c,e). Figure 2g depicts data on root Na^+^ content under −K and −K −P treatments obtained from all plant species under study, including tomato and Arabidopsis, with respect to the root Na^+^ content under −K and −K −P treatments. Tomato plants were the species where the highest increase in root Na^+^ content due to the −K −P treatment was observed (highest distance from the lack‐of‐response line, dashed line). Besides tomato, *C. quinoa* and *H. vulgare* also showed a positive response to the −K −P treatment, indicating that such a response was conserved in at least two plant species (Figure 2g).

**Figure 2:**
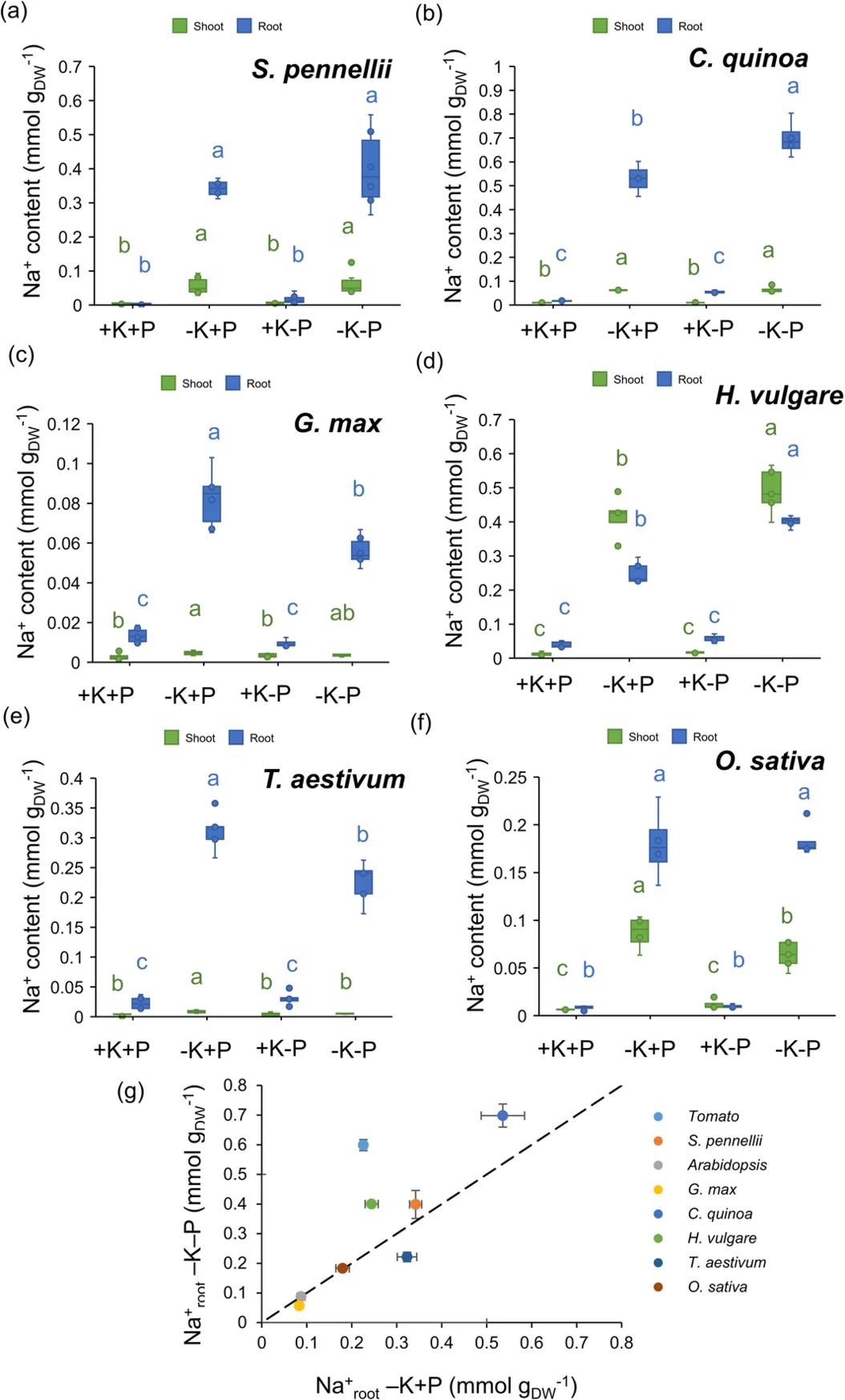
Enhanced Na^+^ accumulation under combined K^+^ and P deficiencies is present in *C. quinoa* and *H. vulgare*. Na^+^ content in roots and shoots of (a) *S. pennelli* (b) *C. quinoa* (c) *G. max* (d) *H. vulgare* (e) *T. aestivum* and (f) *O. sativa* under different combinations of K^+^ and P treatments. (g) Root Na^+^ content under −K −P treatment for the different analyzed species relative to the root content under −K + P treatment. Dashed line indicates a ratio 1:1 in the Na^+^ content in −K −P with respect to the −K + P treatment (no induction by the combined nutrient deficiencies). Plants were grown hydroponically in full nutrient solution for 14 days and then subjected to the aforementioned treatments for 7 days. The external Na^+^ concentration in all solutions was 90 µM. Data are presented in boxplots (*n* = 4–7). Letters represent different groups of data at *p* < 0.05 based on LSD's post hoc test following ANOVA. [Color figure can be viewed at wileyonlinelibrary.com]

### 3.3 The −K −P Treatment Induces a Na^+^ Accumulation Pathway That Exhibits CIPK23‐Dependent and Independent Components in Tomato Roots

Previous results with tomato plants have shown that CIPK23 is a central regulatory component of Na^+^ uptake in K^+^‐starved tomato plants (Amo et al. 2021). Thus, we used the *cipk23* mutant to dissect the contribution of this kinase to Na^+^ uptake in −K −P tomato plants. In the following experiment, WT Micro‐Tom and *cipk23* tomato plants were subjected to −K + P and −K −P treatments for 7 d at an external Na^+^ concentration of 90 µM and the Na^+^ content in plant tissues was determined. As in previous experiments, shoot Na^+^ content was low in both genotypes (Figure 3). Regarding roots, *cipk23* plants showed a 55% reduction in Na^+^ content compared with WT tomato plants in the −K + P solution as described earlier (Amo et al. 2021, 2024) (Figure 3). In the −K −P solution, root Na^+^ content increased in *cipk23* plants with respect to the −K + P solution, but it was still 50% lower than root Na^+^ content of WT tomato plants (Figure 3). Thus, both CIPK23‐dependent and independent components contribute to root Na^+^ accumulation under −K −P conditions.

**Figure 3:**
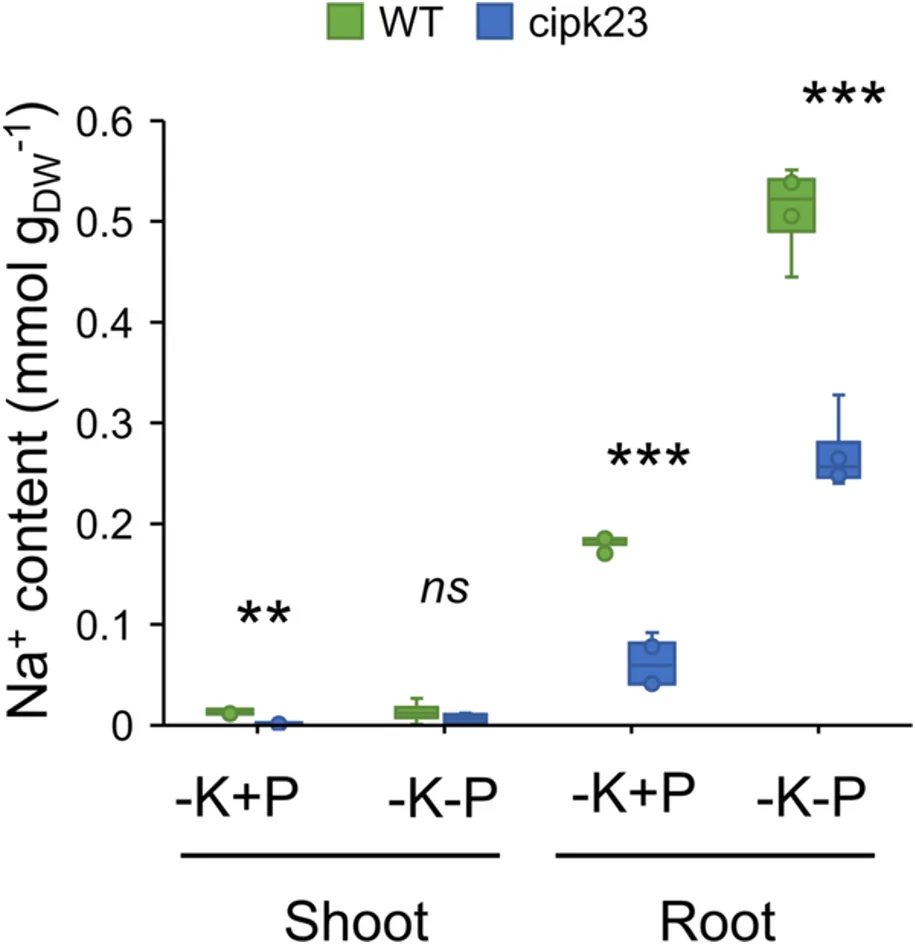
The –K –P treatment induces a Na^+^ transport pathway that exhibits CIPK23‐dependent and independent components in tomato plants. Na^+^ content in roots and shoots of WT and *cipk23* tomato plants. Plants were grown hydroponically in full nutrient solution for 14 days. Subsequently, plants were subjected to K^+^ deficiency (−K + P) or combined K^+^ and P deficiency (−K −P) for 7 d. The external Na^+^ concentration in all solutions was 90 µM. Data are presented in boxplots (*n* = 4). Letters represent different groups of data at *p* < 0.05 based on LSD's post hoc test following ANOVA. [Color figure can be viewed at wileyonlinelibrary.com]

### 3.4 Na^+^ Accumulation Under −K −P Conditions is not Related to P Uptake

Na^+^ accumulation was enhanced by the double deficiency of K^+^ and P and not by deficiencies of other anions (N or S), indicating something specific to the P deficiency. One possibility was that Na^+^ uptake contributed to taking more P up from the external solution when its concentration was very low. A Na^+^/Pi symport mechanism has been described in the marine plant *Zostera marina* (Rubio 2005). Therefore, we explored whether a similar transport system was operating in tomato plants.

In a first experiment, WT tomato plants were grown under standard (0.2 P, 0.2 mM Pi) and low P conditions (0.025 P, 0.025 mM Pi) for the last 7 days of culture. In addition, a subgroup of plants was starved of K^+^ under standard and low P supplies to stimulate Na^+^ accumulation. Regarding the P content in shoots, K^+^ starvation reduced P content under standard and low P conditions (Figure 4a). With respect to roots, growth of plants under low P gave rise to lower P content than plants grown in the 0.2 P solution, but the K^+^ starvation treatment did not affect P content (Figure 4a). Importantly, the higher Na^+^ content observed in −K plants was not associated with a higher P content in the plant with respect to +K plants under standard or low P conditions (Figure 4b). Therefore, Na^+^ accumulation did not improve P accumulation in tomato plants.

**Figure 4:**
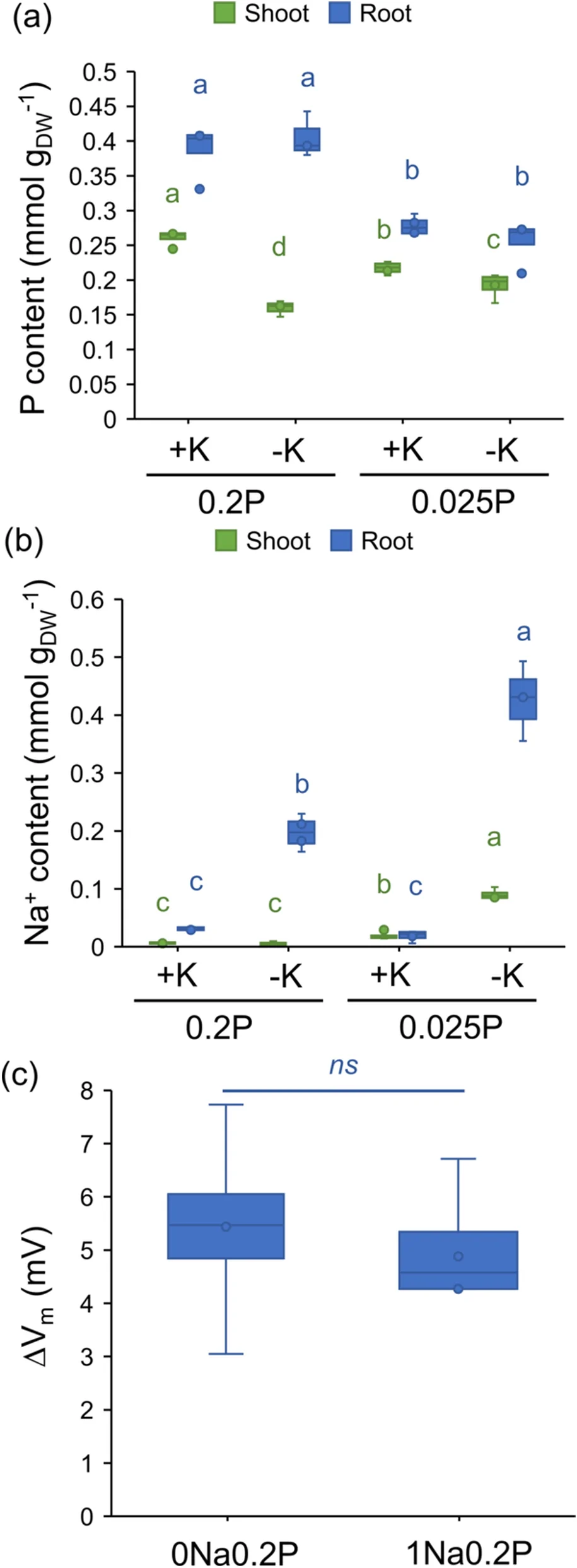
Na^+^ accumulation under –K –P conditions is not related to P uptake. (a) P content in roots and shoots of WT tomato plants. (b) Na^+^ content in roots and shoots of WT Micro‐Tom plants. Plants were grown under standard (0.2 P, 0.2 mM Pi) and low P conditions (0.025 P, 0.025 mM Pi) for the last 7 days of culture. In addition, a subgroup of plants was starved of K^+^ (‐K) under standard and low P supplies. Data are presented in boxplots (*n* = 4). Letters represent different groups of data at *p* < 0.05 based on LSD's post hoc test following ANOVA. (c) Membrane depolarisations of root cortex cells induced by 0.2 mM Pi in the presence (1Na0.2 P) or absence (0Na0.2 P) of 1 mM Na^+^. Root cell impalements were carried out with single microelectrodes on roots of *cipk23* mutant plants grown in –K –P solution for 7 days. Data are presented in boxplots (*n* = 4). A t‐Student test shows no significant differences (ns) (*p* > 0.05). [Color figure can be viewed at wileyonlinelibrary.com]

As an alternative approach, single electrodes were used to measure the membrane depolarisations of root cortex cells induced by Pi in the presence or absence of Na^+^ in the external solution. This approach was previously used to show at the cell level that Na^+^ stimulated Pi uptake in *Z. marina* (Rubio 2005). In our experiments, *cipk23* mutant tomato plants grown in −K −P solution for 7 d were used as they lack the main component of Na^+^ uptake under −K conditions while they retain the −K −P‐induced component of Na^+^ uptake (Figure 3). Thus, the Na^+^ uptake pathways, that do not depend on P starvation but on CIPK23, are absent and there will be less background Na^+^ uptake that may interfere with a putative Na/Pi symport system. In this context, membrane depolarisations of 5.5 mV were induced by 0.2 mM Pi in root cortex cells without added Na^+^ in the external solution (0Na0.2 P solution) (V_m_ before adding 0.2 mM Pi = −147 ± 1 mV) (Figure 4c). When 1 mM Na^+^ was present, the depolarisation induced by 0.2 mM Pi (1Na0.2 P solution) was of 4.6 mV (V_m_ before adding 0.2 mM Pi = −137 ± 1 mV), showing no significant difference with respect to the depolarisation value of the 0Na0.2 P solution (Figure 4c). Thus, Na^+^ did not stimulate Pi uptake in root cortex cells. Overall, Na^+^ uptake in −K −P tomato plants is not employed to improve Pi uptake and should serve other physiological purposes.

### 3.5 −K −P Conditions Upregulate *HKT1;1* in Tomato Roots

In tomato, there are two HKT channels which showed Na^+^ permeability (Asins et al. 2013; Almeida et al. 2014; Amo et al. 2024). Thus, they are relevant candidates to mediate Na^+^ uptake under −K −P conditions. We wanted to explore whether there was a relationship between *HKT* gene expression and the accumulation of Na^+^ in tomato plants. For this purpose, RT‐qPCR was performed on WT Micro‐Tom plants grown for 7 days under +K +P, −K +P, +K −P and −K −P conditions. Regarding *HKT1;1*, the gene was upregulated by single nutrient deficiencies (−K + P, +K −P treatments) compared with +K + P plants (Figure 5a). The −K −P treatment gave rise to a further increase in *HKT1;1* expression, showing that K^+^ and P deficiencies had an additive effect on *HKT1;1* upregulation in the −K −P treatment (Figure 5a). Regarding *HKT1;2*, the gene is downregulated by −K treatment (Figure 5b). However, −P treatment enhanced the expression of *HKT1;2* compared with +K + P plants (Figure 5b). It is worth to note that *HKT1;2* expression in −K −P plants was as high as the −P ones, indicating that the −P treatment overrode the −K effect on *HKT1;2* expression.

**Figure 5:**
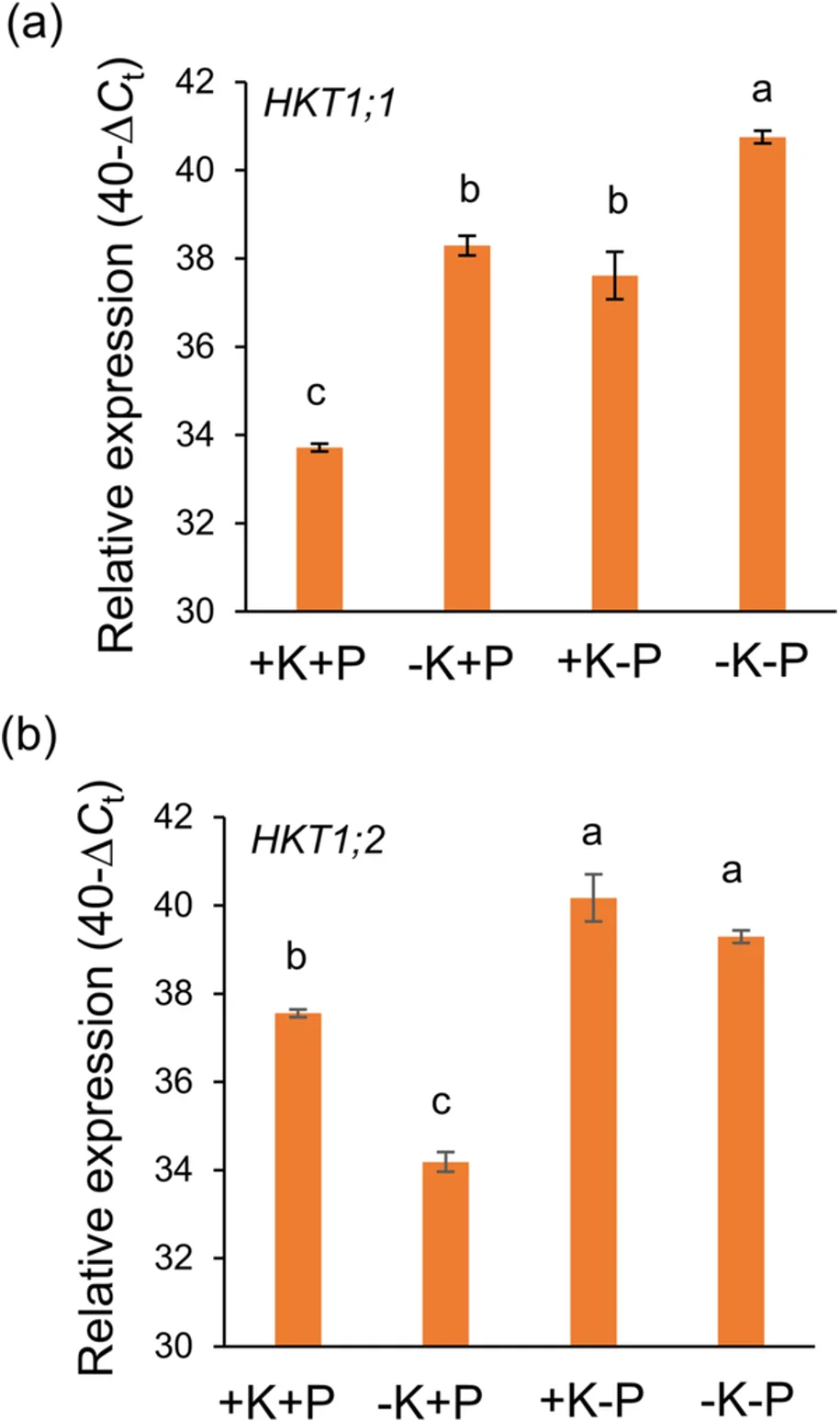
*HKT1;1* and *HKT1;2* are upregulated by –K –P conditions in tomato roots. (a) Relative expression of *HKT1;1* in roots of plants grown under +K +P, −K +P, +K −P and −K −P conditions. (b) Relative expression of *HKT1;2* in roots of plants grown under the conditions indicated in (a). Plants were first grown in 1/5 Hoagland solution for 7 days and later grown for another 7 days under +K +P, −K +P, +K −P and −K −P conditions. qRT‐PCR was carried out on the cDNA using specific primers targeting either *HKT1;1 or HKT1;2*. Gene expression was quantified using the 40− ΔCt method. Data are presented as mean values ± SE (*n* = 3). Letters represent homogeneous groups of data at *p* < 0.05, according to the LSD post‐hoc test following ANOVA. [Color figure can be viewed at wileyonlinelibrary.com]

### 3.6 HKT1;1 Mediates Na^+^ Uptake Under −K +P and −K −P Conditions in Tomato Plants

Given the expression results about tomato *HKTs* and nutrient deficiencies, the next step was to assess whether their function was relevant for Na^+^ accumulation under nutrient deficiency conditions. Single and double RNAi‐silencing lines for *HKT* genes were obtained previously in the NIL14 genomic background (Jaime‐Pérez et al. 2017; Romero‐Aranda et al. 2021). NIL14 is a near isogenic line identified in a segregating population of the Recombinant Inbred Line 157 (RIL 157), which, in turn, was obtained from a cross between *S. lycopersicum var. Cerasiforme* and *S. galapagense* (Asins et al. 2013, 2022). NIL14 possesses the alleles of *HKT* genes from *S. galapagense* in homozygosis, while most of the genome corresponds to *S. lycopersicum var. Cerasiforme* (Asins et al. 2013, 2022). Thus, we conducted an experiment where *HKT1;1 HKT1;2* single and double silenced plants were grown in −K and −K −P conditions at an external Na^+^ concentration of 90 µM as described in Figure 3. Then, Na^+^ and K^+^ contents were determined in these plants. Regarding Na^+^ content, NIL14 control plants exhibited comparable values (Figure 6a) to those shown by Micro‐Tom WT plants under −K + P and −K −P conditions (Figure 1b). For example, values of 0.24 mmol g_DW_^−1^ and 0.64 mmol g_DW_^−1^ in NIL14 control plants under −K +P and −K −P conditions, respectively, and 0.25 mmol g_DW_^−1^ and 0.5 mmol g_DW_^−1^ in Micro‐Tom under −K +P and −K −P conditions, respectively, were obtained in roots. Thus, NIL14 and Micro‐Tom plants responded similarly to the −K −P treatment in terms of Na^+^ accumulation. With respect to the function of HKT channels, *HKT1;2*‐silenced plants exhibited similar values to NIL14 control plants in all conditions and tissues except for −K −P shoots (Figure 6a). However, *HKT1;1* single and *HKT1;1* and *HKT1;2* double‐silenced plants showed lower Na^+^ content than NIL14 plants in all conditions and tissues analyzed (Figure 6a). Such reduction was particularly evident in −K −P roots where a 40‐45% reduction in Na^+^ content was observed in *HKT1;1* single and *HKT1;1* and *HKT1;2* double‐silenced plants (Figure 6a). Thus, *HKT1;1*, but not *HKT1;2*, contributes to Na^+^ uptake under both −K +P and −K −P conditions. K^+^ content data reported no differences among all plant lines and tissues (Figure 6b). Therefore, the contribution *HKT1;1* to Na^+^ uptake was K^+^‐independent. To gain insights into which transport systems mediate Na^+^ accumulation in absence of HKT1;1, the effect of a high external Ca^2+^ concentration (10 mM) on Na^+^ accumulation was tested in NIL14 and *HKT1;1*‐silenced plants. Non‐selective cation channels (NSCCs), which can transport Na^+^, are inhibited if Ca^2+^ is supplied in the millimolar range (Demidchik and Maathuis 2007). Thus, NIL14 and *HKT1;1*‐silenced plants were grown for 4 days in −K −P conditions in the presence or absence of 10 mM CaCl_2_. Relative Na^+^ content in roots (percentage of Na^+^ content relative to −K −P conditions) of NIL14 was not significantly reduced due to the effect of Ca^2+^ (Figure 6c). However, *HKT1;1*‐silenced plants exhibited a 15% reduction in the relative root Na^+^ content in the presence of 10 mM Ca^2+^ with respect to −K −P *HKT1;1*‐silenced plants (Figure 6c). Thus, a Ca^2+^‐sensitive component for Na^+^ accumulation was present in *HKT1;1*‐silenced, but not in NIL14 plants, suggesting the involvement of NSCCs in Na^+^ accumulation in *HKT1;1*‐silenced roots.

**Figure 6:**
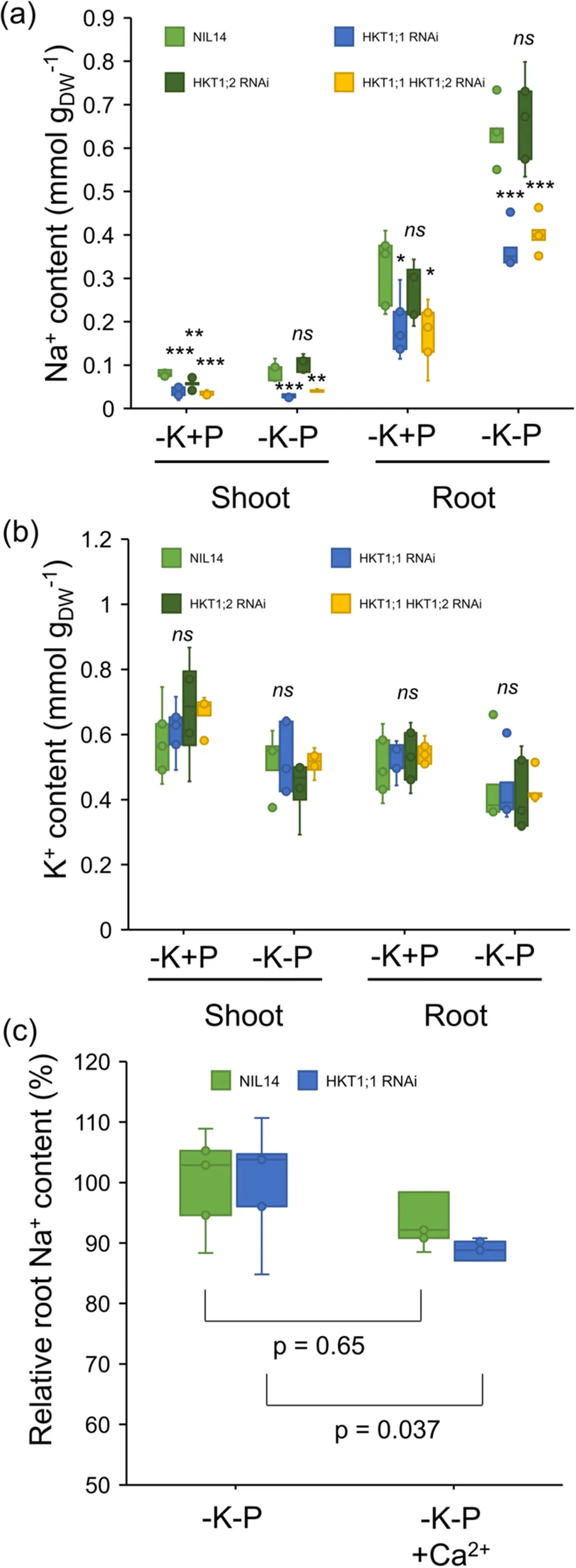
HKT1;1 mediates Na^+^ uptake under −K +P and −K −P conditions in tomato plants. (a) Na^+^ content in roots and shoots of NIL14, *HKT1;1* RNAi, *HKT1;2* RNAi and *HKT1;1 HKT1;2* RNAi plants. (b) K^+^ content in roots and shoots of NIL14, *HKT1;1* RNAi, *HKT1;2* RNAi and *HKT1;1 HKT1;2* RNAi plants. Plants were first grown in 1/5 Hoagland solution for 7 days and then grown for another 7 days under −K +P and −K −P conditions were grown under −K and −K −P conditions as described previously. (c) Na^+^ content of NIL14 and *HKT1;1* RNAi plants starved of K^+^ and P for 4 days in the presence or absence of 10 mM CaCl_2_. (d) Relative root Na^+^ content of the plans shown in (c). The external Na^+^ concentration in all solutions was 90 µM. Data are presented in boxplots (*n* = 5). ** and *** indicate *p* < 0.01 and *p* < 0.001 in Student's *t* test, respectively. Letters represent homogeneous groups of data at *p* < 0.05, according to the LSD post‐hoc test following ANOVA. Root and shoot values were studied in the ANOVA separately, giving lowercase letters for shoots and uppercase letters for roots. In (d), *p* value in Student's *t* test is shown. [Color figure can be viewed at wileyonlinelibrary.com]

### 3.7 *HKT* Promoters Show Variability in the Number of P1BS Motifs in *Solanum* Plants

P1BS motifs (GNATATNC sequence) are well known to be located in promoters of P‐responsive genes (Sobkowiak et al. 2012). Interestingly, there are four P1BS boxes in the *HKT1;1* and one box in the *HKT1;2* promoter regions (from ATG codon to −3 Kb) in both Micro‐Tom and Heinz reference genomes (Supporting Information Figure S3). Thus, part of the gene expression regulation of *HKT1;1* and *HKT1;2* by P starvation (Figure 5) may be due to the presence of such regulatory motifs. After exploring the promoter regions of *HKT* genes in other *Solanum* plants, *S. lycopersicum* var. Cerasiforme and *S. galapagense* (the genetic donors of NIL14 plants, Figure 6) (Asins et al. 2013) had the same number of P1BS boxes as *S. lycopersicum* and showed the −K −P Na^+^ accumulation response, which was dependent on HKT1;1 function (Figure 6a). However, P1BS boxes are missing in such genomic regions in *S. pennellii* (Supporting Information Figure S3a) and this species failed to enhance Na^+^ accumulation under −K −P conditions (Figure 2a). Regarding other *Solanum* species, potato has one P1BS box in the only *HKT* gene it has, whereas *S. lycopersicoides* has one P1BS box in each of its two *HKT* genes at conserved positions with *S. lycopersicum* (Figure S3a). Given the sequence relationship between promoter regions (Supporting Information Figure S3b), it can be deduced that the increase in the number of P1BS boxes in the *HKT1;1* promoter region occurred recently in the tomato evolution. Additionally, we carried out a similar analysis on the *HKT* promoter regions in Arabidopsis, rice, soybean, quinoa, wheat and barley (Supporting Information Figure S4). A large variability in the number and position of P1BS motifs was observed in the *HKT* promoters analyzed. Taking into account the Na^+^ accumulation response to either −P or −K −P treatments in the aforementioned species (Figures 1 and 2), it cannot be deduced that the presence of P1BS motifs in *HKT* promoters was associated with higher Na^+^ accumulation under −P or −K −P treatments, except for barley (Figure 2d and Supporting Information Figure S4f). Four barley *HKT* promoter regions contained P1BS motifs (Supporting Information Figure S4f) and barley plants accumulated more Na^+^ under −K −P treatments than −K plants (Figure 2d). Despite quinoa plants showed a higher Na^+^ content under −K −P conditions with respect −K conditions (Figure 2b), no P1BS motifs were found in *HKT* promoter regions (Supporting Information Figure S4c). A phylogenetic analysis of all the *HKT* promoter regions screened for P1BS motifs evidenced that promoters fell into two large phylogenetic groups (Supporting Information Figure S4g). We expect to gain insights into this topic in the future.

## 4 Discussion

In dicot species, the function of HKT channels is related with salinity tolerance (Riedelsberger et al. 2021; Li et al. 2025). Retrieval of Na^+^ from the xylem under salt stress is mediated by the only HKT Na^+^ channel in Arabidopsis, AtHKT1;1, and its homologue, HKT1;2, in tomato (Sunarpi et al. 2005; Davenport et al. 2007; Jaime‐Pérez et al. 2017). However, in tomato, there is an additional HKT Na^+^ channel, HKT1;1, and we propose here that this tomato channel has evolved to fulfil a different function: Na^+^ uptake at low external concentrations (Figure 6), similar to the function described for the rice OsHKT2;1 channel (Horie et al. 2007). There is a large diversity in the number of *HKT* genes in dicot species which originated from whole genome duplications and tandem duplications (Li et al. 2025). Thus, in species where more than one *HKT* gene is present in the genome, homologous genes to tomato *HKT1;1* may mediate the Na^+^ uptake observed in dicot species.

Na^+^ uptake at low external concentrations has been observed in multiple plant species under low K^+^ conditions, including dicot and monocot species (Haro et al. 2010) with varying degrees of Na^+^ accumulation among them (Figure 2). However, Arabidopsis was the only species in our experiments that failed to increase tissue Na^+^ content under K^+^ deficiency conditions (Figures 1 and 2). Intriguingly, *Eutrema salsuginea*, an extremophile close relative of Arabidopsis, showed the same behaviour as the model plant (Alemán 2009). At this moment, it is unclear whether Arabidopsis and Eutrema are unique cases or other plant species are unable to increase tissue Na^+^ contents under K^+^‐limiting conditions.

One important point is whether Na^+^ uptake could be mediated by ion channels in tomato plants at low external Na^+^ concentrations. In Supporting Information Figure S1, the membrane potentials recorded in root cortex cells were −151 ± 2 mV and −154 ± 4 mV in −K and −K −P roots, respectively. If we consider a Na^+^ concentration of 90 µM in the external solution (Figure 1), and assume a Na^+^ concentration of 20 mM in the cytosol (Carden et al. 2003; Kronzucker et al. 2006), the plasma membrane potential should be more negative than −138 mV to allow the downhill transport of Na^+^ through an ion channel, which is a condition met by our data.

The positive effect of P starvation in Na^+^ uptake in tomato plants was puzzling at the beginning as there are not previous data on this response in the literature. The first hypothesis was that Na^+^ could be used to drive Pi uptake at low Pi concentrations as it has been shown in *Zostera marina* (Rubio 2005). However, our data did not support such a hypothesis as P contents did not increase when Na^+^ accumulation was enhanced (Figure 4a,b) nor Pi‐induced depolarisations were of higher magnitude in the presence of Na^+^ (Figure 4c). Therefore, the P starvation effect on Na^+^ accumulation should be indirect. K^+^ homoeostasis is one important aspect to take into account here. Although there were not differences in the K^+^ content of −K or −K −P plants (Supporting Information Figure S2a), the expression of the gene encoding the K^+^ transporter *HAK5* was higher in the −K −P condition than in the −K condition in tomato roots (Rubio et al. 2014). The measure of tissue K^+^ content reflects mainly the vacuolar K^+^ content (Leigh and Wyn Jones 1984; Walker et al. 1996), and previous reports showed that *HAK5* expression was not correlated with the tissue K^+^ content in tomato plants (Nieves‐Cordones et al. 2008; Leidi et al. 2010). Thus, we hypothesise that P starvation gave rise to a higher degree of K^+^ deficiency in tomato plants, probably related to a lower cytosolic K^+^ concentration, than the single K^+^ deficiency which required more Na^+^ to be compensated. At least, such Na^+^ accumulation in −K −P plants improved root water content values with respect to −K plants (Supporting Information Figure S2d), which suggested a partial osmotic benefit of Na^+^. In this scenario, Na^+^ uptake mediated by the tomato HKT1;1 is particularly relevant as its gene expression is higher in the −K −P conditions than in the single deficiencies (Figure 5a) and RNAi‐silenced *HKT1;1* plants accumulated 40‐45% less Na^+^ in roots than control plants (Figure 6). The presence of P1BS boxes in *HKT* promoters from *Solanum* plants (Supporting Information Figure S3) may give some clues about the P‐dependence of Na^+^ uptake in other plants. The diversity in the number of P1BS motifs in *HKT* promoters in the *Solanum* plants motivates further studies to elucidate to what extent regulation of *HKT* genes by P deficiency has been an important trait in the recent evolution of *Solanum* species. Despite P1BS motifs are present in *HKT* promoter regions in other plant species used in this study (Supporting Information Figure S4), the relationship between P nutrition and *HKT* expression in such species is not evident at present and deserves further investigation.

Although HKT1;1 is an important actor for Na^+^ uptake at low external concentrations in tomato plants (Figure 6), additional Na^+^ transport systems are operating in parallel due to the evident Na^+^ accumulation in *HKT1;1*‐silenced lines (Figure 6). Such systems may include HAK transporters and NSCCs (Amo et al. 2024). The Ca^2+^‐sensitivity of Na^+^ accumulation in *HKT1;1*‐silenced plants (Figure 6c) argues in favour of the involvement of NSCCs in this process. As stimulation of Na^+^ uptake is observed in −K −P *cipk23* tomato plants with respect to the −K *cipk23* plants (Figure 3), it can be deduced that a CIPK23‐independent component of Na^+^ uptake becomes more active under these conditions. Identification of CIPK23‐independent and –dependent transport systems still requires further research.

We have shown that HKT1;1 is the first transport system involved in Na^+^ uptake at low external concentrations in a dicot species. Given the importance of beneficial Na^+^ uptake for crops (Wakeel et al. 2011; Thorne and Maathuis 2022; Ma et al. 2023; Amo et al. 2024), the identification of molecular mechanisms mediating Na^+^ acquisition at low external concentrations is of great interest to allow crops to tolerate low K^+^ and low K^+^/low P regimes. It is worth highlighting that, in this context, Arabidopsis is a limited model as it lacks beneficial Na^+^ uptake (Figure 1). Therefore, research conducted on crops is particularly pertinent to make advances in this topic.

## Conflicts of Interest

The authors claim that there is no conflict of interest related to this work.

## Supporting information


**Figure S1:** Membrane depolarizations of root cortex cells induced by 1 mM Na^+^ in WT plants grown in −K and −K −P during 7 days. Data are presented as mean values ± SE (*n* = 4). * indicates *p* < 0.05 in Student's t‐test.

**Figure S2:** K^+^ and P content, dry weight and water content of plants subjected to single or combined nutrient deficiencies. (a) K^+^ content in roots and shoots of WT Micro‐Tom plants. (b) P content in roots and shoots of WT Micro‐Tom plants. Plants were grown under full nutrient solution in hydroponics during 14 d. Then, plants were subjected to combined deficiency of K^+^ (‐K) with that of NO_3_^‐^ (‐K ‐N), PO_4_^3‐^ (‐K ‐P) and SO_4_^2‐^ (‐K ‐S). A group of plants grown under full nutrient solution was kept as a control. (c) Dry weight and (d) water content of root and shoots of WT tomato plants grown under individual or combined K^+^ and P deficiencies. The external Na^+^ concentration in all solutions was 90 µM. Data are presented in boxplots (*n* = 4–6). Letters represent different groups of data at *p* < 0.05 based on LSD's post hoc test following ANOVA.

**Figure S3:** Identification of P1BS motifs in the *HKT* promoter regions of *Solanum* plants. (a) Position of P1BS motifs (GNATATNC) in the promoter regions (3 kb upstream of ATG codon) of *HKT1;1* and *HKT1;2* genes in *Solanum* species. (b) Phylogenetic tree of the promoter regions of *HKT1;1* and *HKT1;2* genes in *Solanum* species. The tree was built using the Seaview software with the parsimony method.

**Figure S4:** Identification of P1BS motifs in the *HKT* promoter regions of (a) *Oryza sativa*, (b) *Glycine max*, (c) *Chenopodium quinoa*, (d) *Arabidopsis thaliana*, (e) *Triticum aestivum* and (f) *Hordeum vulgare*. Position of P1BS motifs (GNATATNC) in the promoter regions (3 kb upstream of ATG codon) of indicated genes. (g) Phylogenetic tree of the promoter regions of *HKT* genes. The tree was constructed using Seaview software with the parsimony method. In the case of wheat, only *HKT* genes from genome A have been analyzed.

## Data Availability

The data that support the findings of this study are available on request from the corresponding author. The data are not publicly available due to privacy or ethical restrictions.
